# Ancient origins of low lean mass among South Asians and implications for modern type 2 diabetes susceptibility

**DOI:** 10.1038/s41598-019-46960-9

**Published:** 2019-07-19

**Authors:** Emma Pomeroy, Veena Mushrif-Tripathy, Tim J. Cole, Jonathan C. K. Wells, Jay T. Stock

**Affiliations:** 1 Department of Archaeology, University of Cambridge, Downing Street, Cambridge, CB2 3DZ UK; 2 Department of Archaeology, Deccan College Postgraduate and Research Institute, Yerwada, Pune, 411 006 India; 3 UCL Great Ormond Street Institute of Child Health, University College London, 30 Guilford Street, London, WC1N 1EH UK; 4 ADaPt Project, PAVE Research Group, Department of Archaeology, University of Cambridge, Pembroke Street, Cambridge, CB2 3DZ UK; 5 Department of Anthropology, University of Western Ontario, London, Ontario N6A 5C2 Canada; 6 Department of Archaeology, Max Planck Institute for the Science of Human History, Kahlaische Strasse 10, Jena, Germany, Jena, Germany

**Keywords:** Biological anthropology, Type 2 diabetes

## Abstract

Living South Asians have low lean tissue mass relative to height, which contributes to their elevated type 2 diabetes susceptibility, particularly when accompanied by obesity. While ongoing lifestyle transitions account for rising obesity, the origins of low lean mass remain unclear. We analysed proxies for lean mass and stature among South Asian skeletons spanning the last 11,000 years (n = 197) to investigate the origins of South Asian low lean mass. Compared with a worldwide sample (n = 2,003), South Asian skeletons indicate low lean mass. Stature-adjusted lean mass increased significantly over time in South Asia, but to a very minor extent (0.04 z-score units per 1,000 years, adjusted R^2^ = 0.01). In contrast stature decreased sharply when agriculture was adopted. Our results indicate that low lean mass has characterised South Asians since at least the early Holocene and may represent long-term climatic adaptation or neutral variation. This phenotype is therefore unlikely to change extensively in the short term, so other strategies to address increasing non-communicable disease rates must be pursued.

## Introduction

Non-communicable diseases (NCDs) accounted for 60% of global deaths in 2012 and place a growing burden of morbidity and mortality on populations worldwide^1^. India has been described as a ‘diabetes capital of the world’^2^ due to its large population and their elevated susceptibility to NCDs. In 2017, 10% of Indian adults (73 million people) had type 2 diabetes (T2D), second only to China in absolute terms, and India is projected to rank first by 2045 as population, lifespans and urbanisation increase^3^. Other South Asian countries (broadly Pakistan, Sri Lanka Bangladesh, and Nepal) show a similar emerging disease profile^3,4^. While lifestyle factors (dietary trends, more sedentary lifestyles) and obesity clearly play an important role in NCD susceptibility, inter-population variation is not fully explained by such exposures. Within specific settings, people of South Asian ancestry have an elevated risk of T2D compared with other groups^5,6^. For example, South Asians in London, UK, had 2–3 times greater T2D risk compared with those of European ancestry, with onset typically 5 years earlier and at a lower body mass index (by 5 kg/m^2^)^7^. Here, we investigate the origins of a key factor implicated in this elevated susceptibility: low lean mass. When and why this phenotype originated is currently unknown, and understanding the origins of South Asian low lean mass may have important implications for how we address the growing burden of NCDs in this population.

Contemporary South Asians typically have lower lean mass (organ and muscle mass) relative to stature and total body mass than Europeans^8^, which may partly explain why they develop NCDs at a lower BMI than other populations. South Asian low lean mass is present at birth, and this difference becomes more pronounced after adjusting for their low average birth weight^9^. Neonatal low lean mass persists even four or five generations after migration to other parts of the world including the UK^10,11^, Netherlands^12^ and Surinam^13^, despite changes in diet and environment. This suggests the phenotype is heritable in the broader sense, but whether by genetic or epigenetic mechanisms is unknown. Lower lean mass is associated with lower glucose clearance and possibly earlier beta-cell exhaustion^14^, so is thought to contribute causally to elevated diabetes risk^15^ in the context of obesity. Contemporary South Asians are also generally characterised by relatively short stature^16^, which is associated with lower glucose tolerance independently of body mass^17^ and may act as a marker of T2D susceptibility, including during pregnancy^2,18^.

The temporal and causal origins of low lean mass among South Asians are unknown but various hypotheses have been proposed (reviewed in^2^). Briefly, on the longest timescale, adaptation to a predominantly hot, equatorial climate^19^ may have led to selection for lower body mass (which generates less heat and increases heat loss through a greater surface area to volume ratio) to reduce thermal load. The oldest *Homo sapiens* remains from South Asia date to ~38,000 years ago, but modern human occupation of the region may date back even further^20^, demonstrating a long population history in the region. Climatic unpredictability might also have selected for lower lean mass as an adaptation to unreliable food resources (though this does not equate with small being ‘healthy’ - see^21^). South Asia is affected by the unpredictable El Niño Southern Oscillation (ENSO), causing an erratic resource base. During the first half of the Holocene, the ENSO phenomenon was much less frequent or absent, while the current ENSO pattern was established about 5,000 years ago^22^.

The transition from hunting and gathering to food production may also have selected for smaller lean mass and stature among South Asians. Height decreased in many parts of the world with the agricultural transition^23,24^ including South Asia^25,26^, as populations became more vulnerable to famine, seasonal shortages, nutrient deficiencies and infectious diseases associated with sedentary communities^23^. The transition to food production in South Asia began broadly around 9,000 years before present (BP) in the north west of the region and spread south and east, reaching the extreme south by ~4,600 years BP^27^. The more recent adoption of vegan or vegetarian diets may also be implicated in the ontogenetic development of low lean mass^28^. Such diets are widespread today in South Asia, and may date back to at least the third century BCE according to documentary evidence^29^, although archaeological evidence suggests that meat consumption was widespread until a few centuries ago^29,30^. On the shortest timescale, societal pressures in the context of unpredictable ecological conditions may explain the South Asian phenotype. Repeated, severe famines affected South Asia in the 19^th^ and first half of the 20^th^ centuries, which were exacerbated by British colonial policy and were associated with high mortality from starvation^31^. This might have selected for genes associated with low lean mass, or might have reduced lean mass through mechanisms of trans-generational plasticity^2^.

**Figure 1:**
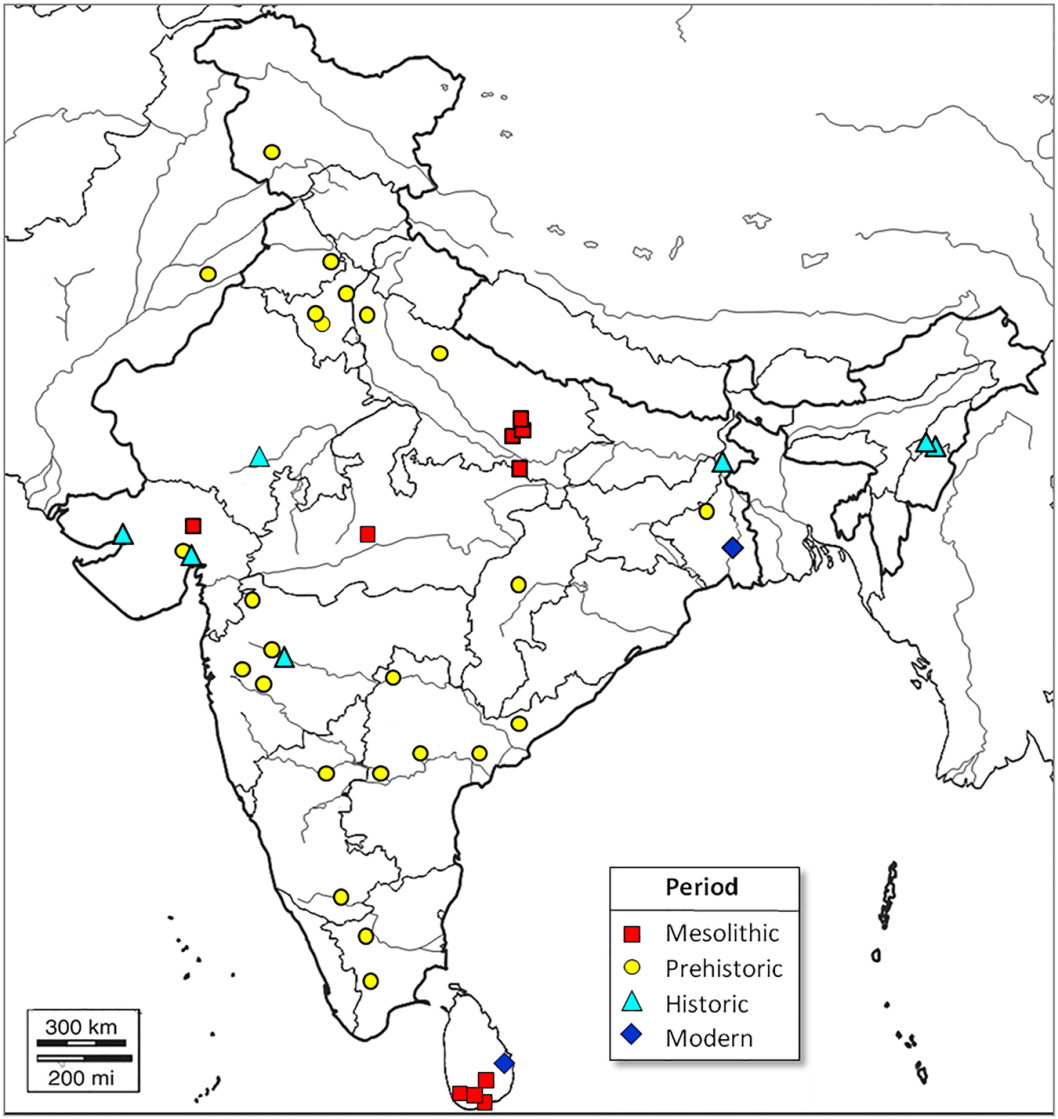
Map of South Asia showing location of study samples.

**Figure 2:**
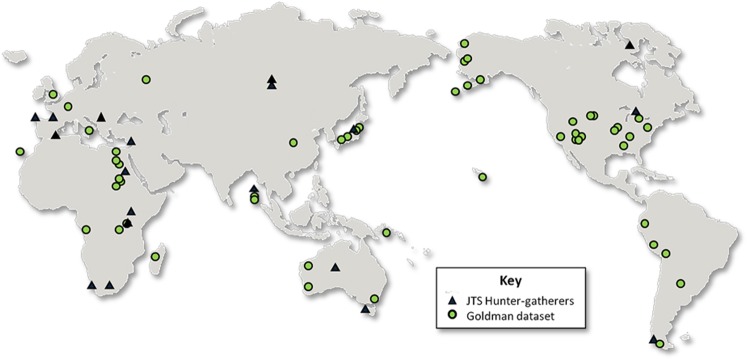
World map showing location of comparative Late Pleistocene and Holocene data used in this study.

Here, we investigate the temporal origins of South Asian low lean mass by inferring trends in relative lean mass and stature over the last 11,000 years using archaeological and recent South Asian adult skeletons (n = 197, Fig. 1: sufficiently preserved skeletons predating 11,000 years BP are yet to be discovered in South Asia). We use skeletal proxies for lean mass and stature in the context of variation among a global sample of terminal Pleistocene and Holocene adult *Homo sapiens* skeletons (n = 2,003, Fig. 2). We hypothesise that if low lean mass is a long term climatic adaptation it will be evident throughout the last 11,000 years, while if it originates from more recent dietary change or societal pressures, a change in relative lean mass should be expected that coincides temporally with these events. Understanding the timing of the origin of low lean mass and shorter stature among South Asians may offer novel insights into the relative impacts of the natural and social environments on human body size, growth and health, and have implications for devising effective strategies to tackle the growing burden of NCDs in this population^32^. If low lean mass is a recent characteristic, it may recover over the next few generations, while a long term adaptation will take longer to reverse, so the two have different implications for devising effective preventive strategies^32^.

## Results

### Inter-population variation in inferred lean mass relative to stature

**Figure 3:**
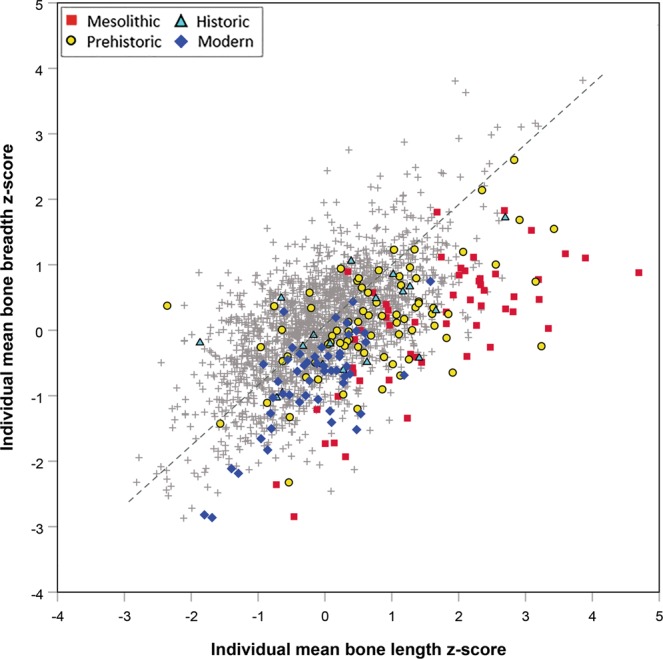
Plot of individual mean bone breadth z-score against individual mean bone length z-score in South Asian archaeological and modern skeletons from the last 11,000 years (n = 197) compared with a worldwide sample of terminal Pleistocene and Holocene humans (n = 2,003: grey crosses) demonstrating that South Asians throughout the study period typically have low lean mass (bone breadth z-score) relative to stature (bone length z-score). Reduced major axis regression line fitted to the whole dataset shown as grey dashed line.

Relative to stature (indicated by mean bone length z-scores for each individual) South Asian skeletons demonstrate low lean mass (low individual mean bone breadth z-scores) compared with other worldwide populations, since most individuals fall below the reduced major axis (RMA) regression line for the total dataset (Fig. 3). Considering the South Asian data by broad time periods, the Mesolithic hunter gatherers typically have higher length z-scores than more recent South Asians (i.e., taller stature), but in all groups, individuals have relatively low breadth z-scores in relation to length z-scores, indicating that low lean mass relative to stature characterised South Asians throughout the past 11,000 years.

### Temporal trends in South Asian lean mass

**Figure 4:**
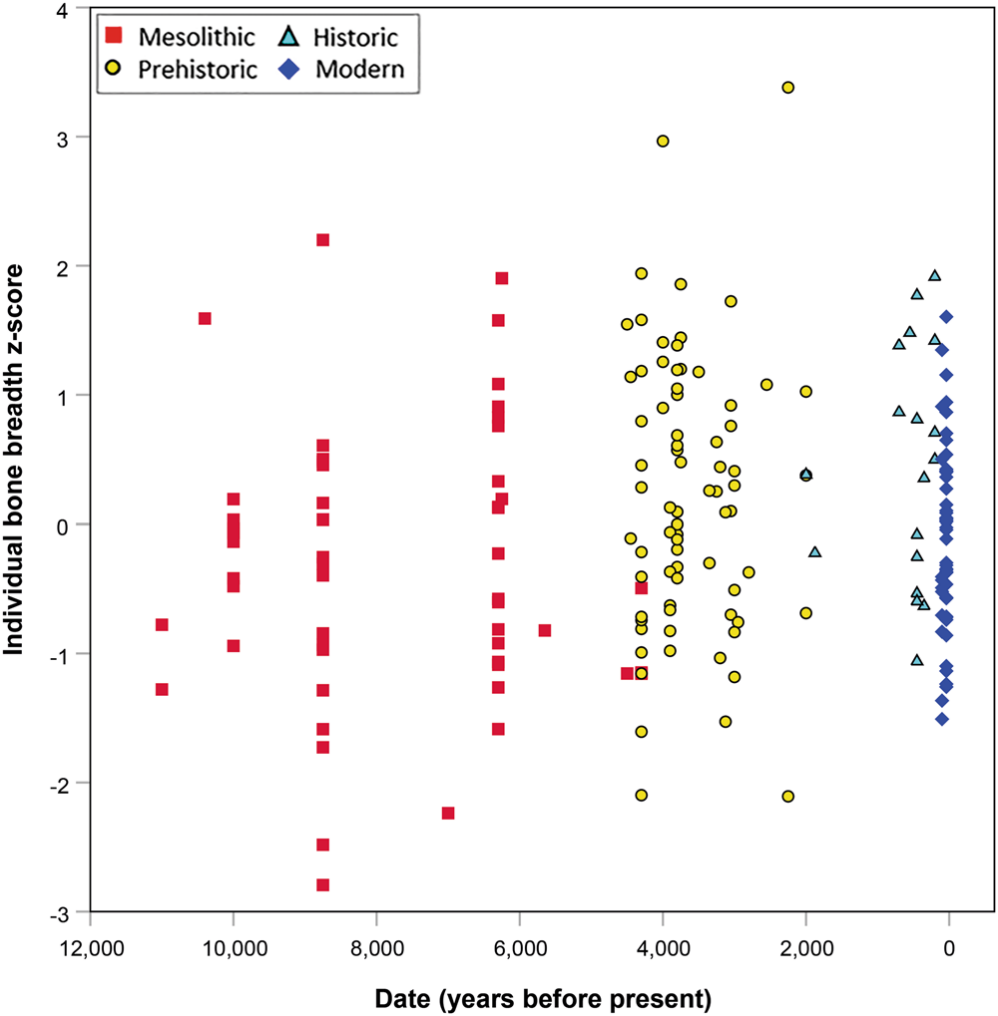
Individual mean bone breadth z-score (adjusted for latitude and bone length z-score) plotted against date of site, illustrating a minor temporal trend in relative lean mass among South Asians (n = 197) over the last 11,000 years.

In the South Asian dataset, breadth z-score adjusted for length z-score and latitude (to account for geographic patterning in the data) showed a slight significant increase through time (Fig. 4, Supplementary Table 1: trend for date in linear regression model, p = 0.02), indicating that there was an increase in relative lean mass of South Asians over this period, albeit small in magnitude (0.04 z-score units per 1,000 years, standard error = 0.02), and explaining only 1% of the variance in bone breadth z-score.

### Temporal trends in South Asian stature

**Figure 5:**
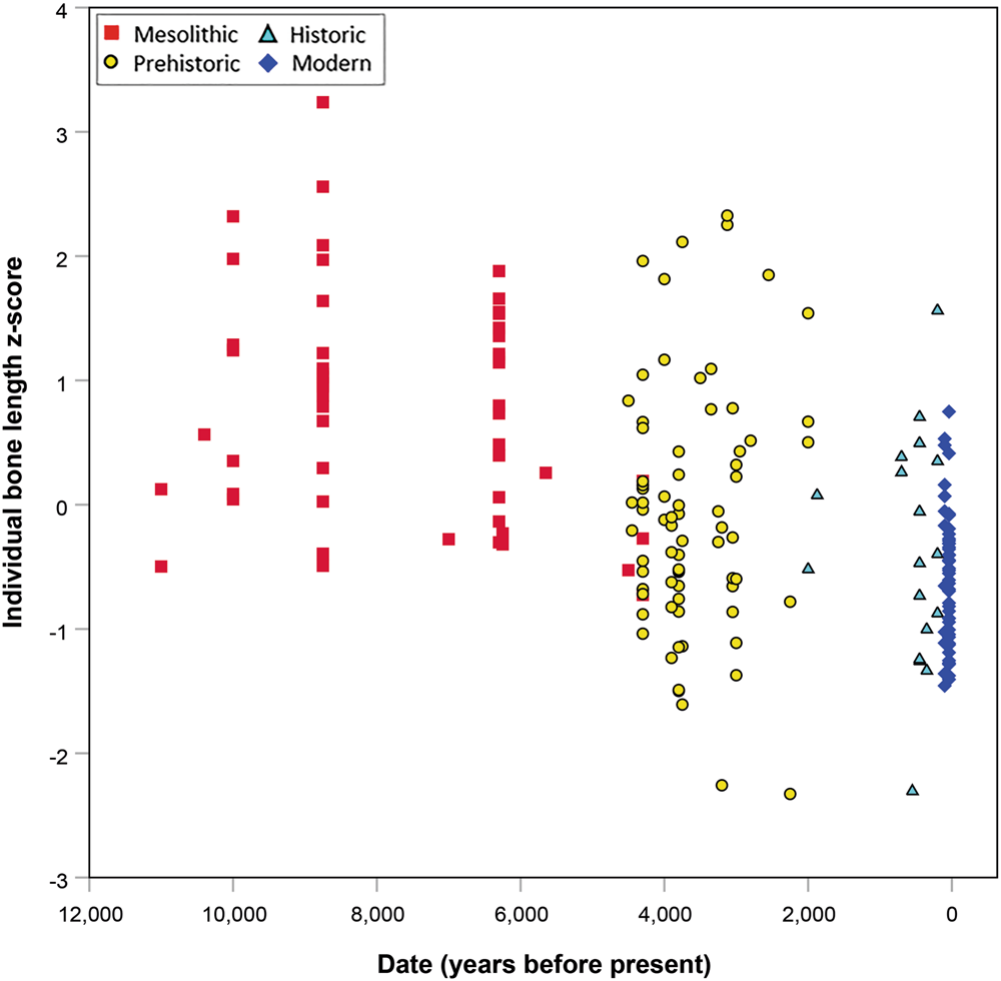
Individual mean bone length z-score (adjusted for latitude) plotted against date of site, illustrating temporal trends in inferred stature among South Asians (n = 197) over the last 11,000 years. There is a major decrease between the Mesolithic and Prehistoric samples (n = 54 and 75 respectively), followed by a slower decline from the Prehistoric period through to the 20^th^ century.

Stature (length z-score) fell by 1.2 units (p < 0.001) between Mesolithic hunter-gatherers and all later populations once agriculture was adopted (Fig. 5). This step change was followed by a slower, linear decline from 5,000 years BP up to the 20^th^ century. Restricted to the last 5,000 years, regression of bone length z-score on date, adjusting for latitude, indicated a decline of 0.22 (standard error = 0.05) z-score units per thousand years (adjusted R^2^ = 0.12, p < 0.001). To put this in context, the standard deviations of femur length are 30.1 mm and 26.5 mm for males and females respectively, and using stature prediction equations^33^, one z-score difference in femur length equate to 7.5 cm and 7.0 cm difference in estimated stature for males and females respectively. Thus we might predict total average declines in stature from Mesolithic hunter-gatherers to later populations of 8.5 cm and 7.7 cm in males and females respectively, and a decline of the same magnitude across the 5,000 years since agriculture was adopted.

## Discussion

This analysis of South Asian adult skeletal material demonstrates that compared with a worldwide sample of terminal Pleistocene and Holocene skeletal variation, South Asians have persistently low bone breadth z-score relative to length z-score, indicating relatively low lean mass for stature. Bone breadth z-score (adjusted for length z-score and latitude) among South Asians showed statistically significant increase through time, albeit of very small magnitude and explaining less than 1% of the variation, so relative low lean mass appears to have been a constant characteristic of South Asians across the last 11,000 years. Adjusted for latitude, South Asian bone length z-scores decreased between the Mesolithic and later periods by 1.2 z-scores, indicating a marked fall in stature with the adoption of agriculture, followed by a more gradual decline in stature through to the 20th century.

Our finding that South Asian low lean mass has ancient origins would be most consistent with long-term adaptations to ecological pressures, rather than more recent dietary change or the impacts of 19^th^–20^th^ century famines exacerbated by British colonial policy. The lack of well-preserved postcranial remains predating 11,000 years BP prevents us from examining earlier trends in South Asian lean mass. Other researchers have noted the relatively “gracile” bones (narrow relative to their length) of Mesolithic South Asians compared with more robust hunter-gatherers^34–36^ and have also attributed this slight build to climatic adaptation^35,36^. Our data show that this characteristic persists into recent times.

Interestingly, skeletal remains of East Africans and native Australians show similar patterns of low bone breadth relative to length, and by inference low lean mass^35^. Like South Asians, native Australians have an elevated incidence of NCDs^37^, relatively low lean mass, a higher proportion of body fat for a given BMI, and a tendency towards abdominal obesity^38,39^, although their relatively long limbs attenuate some of these contrasts^40^. South Asia and Australia were both colonised relatively early by dispersals of modern humans, and both subsequently had long periods (tens of thousands of years) for *in situ* development with relatively low levels of gene flow^41–43^. Whether there is a similar link between low lean mass and T2D susceptibility among South Asians and native Australians, and whether such phenotypic similarities reflect common ecological factors (equatorial climates susceptible to ENSO effects) or potentially neutral processes/shared ancestry could not be addressed here and are questions for future investigation.

Evidence that South Asian low lean mass is strongly heritable might indicate a still-unidentified genetic basis. There is evidence for natural selection near the *Myostatin* (*MSTN* or *GDF-8*) gene among South Asians^44^, which decreases skeletal muscle mass in fetal and postnatal life, but the nature and effect of any changes to this gene in South Asians remain to be clarified. In a sample of north Indian adults, variants at this locus were associated with variability in lean mass and (abdominal) obesity^45^. Alternatively, the heritability of low lean mass may originate from an intense cycle of inter-generational plasticity that is hard to break: low maternal lean mass may be the strongest predictor of low offspring lean mass at birth^46^, and low birth weight (associated with lower lean mass) predicts low adult lean mass^47^. Fifty generations of undernutrition in a rat model led to the development of a similar phenotype (including low birth weight, central adiposity, insulin resistance, and vitamin B12 and folate deficiency) in the absence of genetic change^48^. The phenotype largely persisted for 2 generations after returning the offspring to a standard diet (although birth weight and fat mass did show partial recovery), indicating that the South Asian phenotype might plausibly result from multigenerational undernutrition. Our study is unable to shed light on the heritable basis of low lean mass of South Asians but does indicate that it is a longstanding characteristic.

Other factors are also likely to contribute to elevated NCD susceptibility among South Asians. Genetic loci associated with obesity and/or T2Ds have been identified among South Asians^49,50^, while the impacts of early life environment on later growth and metabolic function may also be partly responsible^51,52^. Low birth weight is particularly common in India^53^ and is associated with reduced ‘metabolic capacity’ in adulthood (including muscle mass, pancreatic beta cell mass, and renal nephron number)^54,55^. Especially under conditions of elevated ‘metabolic load’ (high sugar and fat diets in a context of low activity levels) individuals with lower capacity are more susceptible to obesity and associated NCDs^56^. All these factors appear to link low lean mass and excess adiposity to NCD risk, and differ on the timescales on which they operate^15^.

A key component of NCD susceptibility is excess adiposity. Unfortunately, at present there are no methods to reliably estimate body fat from skeletal dimensions^57–59^ so we were unable to investigate temporal trends in fat mass alongside lean mass. Since fat mass is highly plastic, contingent on local environment, age, diet and activity, and largely a result of modernisation, this component of NCD risk is a distinct phenomenon. Lean mass is low in South Asians compared with other populations across the range of BMI^8,60^ i.e., regardless of obesity status, and thus patterns of lean mass variation are of greater importance for understanding the origin of baseline disease susceptibility.

Unlike the temporal stability in relative lean mass, the marked decrease in mean long bone length z-scores (proxies for stature) between the Mesolithic and subsequent periods coincides with changing patterns of niche construction associated with agriculture, and echoes other studies of South Asia^25,26^ and other parts of the world^23,24^. This decline in stature in South Asia following the adoption of food production is estimated elsewhere to be ~9 cm in males^26^, similar to our results. The decline in stature with the adoption of agriculture appears to have been particularly marked in South Asia (e.g., compare^24^), although uneven spatial and temporal distribution of the data in different world regions complicates any direct comparison. Decreased stature may indicate an additional increase in underlying NCD susceptibility among South Asians on top of that resulting from low lean mass. Short stature is thought to be associated with T2D susceptibility because, like low lean mass, it is linked to poor early life conditions (potentially across multiple generations), which are also known to increase NCD susceptibility^18,61^. Interestingly, the relationship between T2D risk and stature is reportedly strongest in Asians and native Australians compared to other populations^62^.

Although Mesolithic South Asians were generally tall, 4 out of the 5 Sri Lankans, one individual from Damdama and one from Deulga Hills, India^63^ (the latter could not be included in this study due to poor preservation), had very low bone length z-scores before latitude adjustment. This may indicate some interesting variation in hunter-gatherer body size within South Asia, whereby some populations were extremely short while others very tall. The potential causes of this variation require future investigation.

The continued gradual decline since agriculture was adopted is consistent with previous analyses of skeletal and anthropometric data^25,64^ and may reflect the ongoing impact of agriculture, exacerbated by more recent societal pressures in a context of severe famines. The limited size and diversity in geography and social status of available skeletal samples for South Asia means there may have been more recent, shorter-term variation in stature and lean mass that we were unable to detect. Therefore the transition to food production or repeated famines may still have influenced relative lean mass in South Asian populations. Nonetheless, unlike the clear decrease in stature with the adoption of food production, relative lean mass does not appear to have altered significantly over the last 11,000 years. This pattern, whereby stature appears to relate more to nutritional factors, while physique (bone breadth and body mass) appears to reflect ecological (climatic) pressures, is consistent with theoretical models and empirical data concerning variation in human skeletal size and proportions^65,66^.

A limitation of our analyses is that we could not investigate and control for genetic, environmental and other sources of variation across the extensive geographic region of South Asia, which is widely recognised for its genetic, morphological, linguistic and cultural diversity^67^. Recent studies evaluating large-scale geographic variation in human morphology have incorporated genetic and morphological data to tease apart neutral and adaptive influences on phenotype (e.g.^68,69^). In the case of South Asia, ancient DNA studies have generally failed due to unfavourable preservation conditions, making links between ancient and modern populations in the region uncertain. Therefore any attempt to replicate combined genetic and skeletal morphological approaches, especially in the light of the small and fragmentary archaeological skeletal samples available, is problematic at present. While variation in T2D rates^70^, stature^25,71^ and obesity^72^ across contemporary South Asia has been documented in association with environmental factors (e.g. urban vs. rural location), the contributions of genetic and geographic influences on this variation are not well understood, and variation in low lean mass in this region is poorly documented. Thus, any attempt to relate the archaeological data with this modern variation would be speculative and has not been attempted here. *In vivo* body composition data confirm that low lean mass is widespread across the region today, and as South Asians do share a deep common ancestry (see above), a large scale regional approach is justified. We look forward to the time when improved data will facilitate a more nuanced and detailed analysis of intra-regional variation and its causes.

The use of proxies for stature and lean mass is clearly another source of potential error in the dataset, but one that cannot be avoided given that skeletal remains are our only available data source for prehistoric South Asian phenotypes. The proxies we used are well established (as discussed in the Methods), and the use of z-scores rather than estimation equations avoid adding a further step that could introduce further error. Another limitation of the study is that we combined published and novel data because of limited availability of archaeological collections for new study. While this may have added noise to the data due to inter-observer error in the skeletal measurements, we selected only published data collected using methods comparable to our own to limit this effect. Furthermore, inter-observer error in such measurements is generally low^73^. The preponderance of males in our South Asian samples may have affected the outcomes if any changes in stature or lean mass were stronger in one sex than the other. The use of sex-specific z scores accounts for differences in body size between males and females, and we are not aware of any evidence for sex differences in the relationship between lean mass and type 2 diabetes. Low lean mass characterises contemporary South Asians regardless of sex. It remains possible that patterns of change in lean mass may have differed between the sexes, but the size of the available skeletal sample does not permit us to investigate this question at present. Finally, there are limitations to the accuracy of the sample chronology, as few samples have been directly dated with modern radiometric methods and many rely on older radiocarbon dates calibrated by various methods, or by relative dating using established regional cultural sequences (see Methods for more detail). Again, while this may lead to some noise in the data, the relative age of the different skeletal samples should be robust. All the above factors may have led to more conservative results, but we would not expect them to be sufficient to invalidate our findings (see Methods for further discussion).

In conclusion, our analyses suggest that the low lean mass phenotype characteristic of contemporary people of South Asian ancestry has existed for at least 11,000 years. The available data have insufficient resolution to show whether or not there were further small changes in proportional lean mass since the adoption of food production, or as a consequence of more recent famines. Given the antiquity of this phenotype, the most likely explanations for low lean mass in South Asia appears to be either climatic adaptation or neutral evolutionary processes, but the available data do not allow us to distinguish between these hypotheses at present. Consistent with previous work, our data indicate that stature, which is also associated with elevated T2D susceptibility, fell sharply with the transition to food production, and continued to decline more slowly until the 20^th^ century. The implication of our results is that South Asian low lean mass, and associated NCD susceptibility, may have a genetic basis and is unlikely to change in the short term, so that other strategies (such as behavioural and/or dietary intervention) are required to address the epidemic of NCDs that is particularly acute in South Asia.

## Methods

The sample comprises all adult skeletons for which data could be collected or gathered from publications from archaeological sites or historically-collected specimens from India, Sri Lanka, Pakistan and Bangladesh (n = 197, 59 female and 138 male: see Fig. 1 and Supplementary Table 2). It is unknown whether there is currently geographical variation in the low lean mass phenotype within South Asia. Like many quantitative traits, it is likely to show a clinal distribution rather than distinct geographical limits. While other Asian populations show a tendency towards proportionally lower lean mass, this is less marked than among South Asians^74^. Particularly low lean mass and elevated T2D risk has been reported among geographically distant Pakistani, Bangladeshi, Sri Lankan and Indian populations or people with origins in those countries^10,75^, and although data are sparse, in Indian tribal and caste groups alike^76^. Thus skeletal collections from these countries were sought and included.

VMT and EP measured all available adult skeletons (assessed by full epiphyseal closure with the exception of the medial clavicle, third molar eruption, and/or fusion of the spheno-occipital synchondrosis) curated in the archaeological collections at Deccan College Post-Graduate and Research Institute, Pune (n = 35) and a mean of their measurements was used. Additional data were collected by EP and JTS (n = 2) or from published sources (n = 120) where access to the collections was not possible. Data were added to the database only where we could be confident that the measurement definitions used by the authors matched those used for this study.

Data were also collected by EP (n = 40) from teaching skeletons at the University of Toronto (St George and Mississauga campuses) that are of recent individuals from India. They were obtained in the second half of the 20th century from suppliers in Kolkata (S. Pfeiffer, pers. comm. 2016), similar to teaching collections in numerous US and UK institutions^77^. While such collections can be problematic due to mixing elements from multiple individuals before or after sale, each skeleton was examined to ensure that limb and pelvic bones were consistent with belonging to a single individual through limb proportions and joint congruity, and elements which did not fit or individuals deemed extensively mixed were excluded. Such skeletons were typically obtained from relatively poor, low status individuals^77^. Therefore the sample is unlikely to be phenotypically or genetically representative of the Indian subcontinent in the 20^th^ century. Nonetheless, our results for stature are consistent with previous analyses of long-term trends in South Asian stature based on estimates from archaeological skeletons and modern anthropometric data^25,26,64^. Given the care taken in measuring and assessing the integrity of modern skeletons used in our study and concordance with previous work, we can be confident that our findings are broadly reliable.

The combination of measurements from multiple observers (including from publications) raises questions concerning inter-observer error on the data. We selected published measurements carefully to ensure they were taken in an equivalent manner to ours, but we are unable to quantify the magnitude of inter-observer error in our final dataset. Previous research suggests postcranial measurements can be relatively reliable across different observers^74^, though any inter-observer error will have resulted in more conservative results.

To put the data in the context of worldwide skeletal variation we used a dataset of Late Pleistocene and Holocene adult skeletons (n = 2,003: Fig. 2) composed of the Goldman Dataset (n = 1,527)^78,79^ and a database of global hunter-gatherer skeletal data collected by JTS (n = 476: Fig. 2). Climatic influences on preservation, a relatively limited density of archaeological excavations, and past mortuary practices (widespread cremation) limit the availability of data from South Asian archaeological skeletons. Consequently, to maximise sample size we used both long bone joint and shaft breadths as proxies for lean mass and long bone lengths as proxies for stature from all available major limb bones (Table 1). Measurements were selected based on availability in published sources and comparative datasets.

**Table 1:** Skeletal measurements used in the study.

Bone	Lengths	Breadths
Humerus	Maximum (#1)	Supero-inferior head diameter (#10)
		Antero-posterior head diameter*^98^
		Distal epicondylar breadth (#2)
Ulna	Maximum (#1)*	
Radius	Maximum (#1)	
Femur	Maximum (#1)	Supero-inferior head diameter (#18)
		Antero-posterior head diameter (#19)*
		Supero-inferior neck diameter (#15)*
		Subtrochanter medio-lateral diameter (#9)*
		Midshaft medio-lateral diameter (#7)
		Distal epicondylar breadth (#21)
		Distal joint surface breadth^99^
Tibia	Maximum (#1a)	Proximal joint surface breadth (#3)
		Tibia midshaft medio-lateral breadth (#9)
		Distal epiphyseal breadth (#6)

Measurement definitions follow Martin and Saller^97^, Corruccini and Ciochon^98^, and Pearson^99^. * denotes measurements available in JTS’s hunter-gatherer database but not the Goldman Dataset. Numbers in parentheses preceded by ‘#’ refer to the Martin numbers^97^ for the relevant measurements.

Joint surface dimensions, particularly femoral head diameter, are widely used to estimate body mass^79,80^. They do not respond to altered loading due to activity levels or body mass during adulthood, but are widely considered to be fixed at the end of growth at a size proportional to body mass^57,81^, and thus represent what we might consider early adult (or ‘peak’) phenotype. We can expect joint dimensions to act as a reliable proxy for lean mass because: (1) in past populations excess adiposity is assumed to have been uncommon; (2) excess weight gain in more traditional societies typically occurs from middle adulthood^82^ after joint diameters are fixed; (3) lean mass is the major component of total body mass^83^; (4) joint dimensions show a closer relationship to lean mass than fat or total body mass^57,59^; and (5) joint dimensions are unaffected by age at death or changes in lifestyle during adulthood.

Unlike joint dimensions, shaft dimensions respond to changes in loading (both from activity and body mass) during life and are affected by age^84,85^. Activity levels also influence muscularity and thus lean mass, so variation in behaviour could confound estimates of lean mass derived from bone shaft diameters. However, we can still expect bone shaft dimensions to be reliable indicators of lean mass for several reasons. Body mass is the major determinant of long bone cross-sectional properties, accounting for approximately 80% of observed variation^86^. Consequently, analyses of activity patterns adjust shaft cross-sectional properties for body mass^87^ and body mass estimation equations based on cross-sectional geometry have been previously derived for non-adults^88^. Particularly in past, presumably relatively lean and active populations, lean mass would be the major determinant of total body mass. In fact, there is evidence that both joint and shaft diameters are more strongly related to lean mass than to total body mass, and correlate poorly with fat mass^58,59,89^. While these data derive from contemporary populations whose diets and lifestyles likely differ significantly from those of many of the archaeological samples included in our study, the concordance of evidence from diverse samples (European varsity level athletes and controls, rural-urbanising South Asian young adults, and elderly Afro-Caribbean males) would suggest this link between lean mass and bone shaft dimensions is valid. Shaft diameters of both upper and lower limb bones are similarly related to lean mass^58^ justifying the inclusion of bone shaft properties from both limbs. Finally, although bone cross-sections do respond to changes in loading with age, they are thought to mainly reflect loading in late adolescence/early adulthood^84^.

Thus while the use of bone shaft diameters may introduce some noise into our data on inferred lean mass, the benefits of greater sample size from including such measures, given limited skeletal preservation, are likely to outweigh any disadvantage. The evidence suggests that shaft dimensions should still be good indicators of lean mass, and excluding skeletons without joint breadth measurements reduces the sample size from 197 to 139. Nonetheless, to be cautious we repeated all analyses using only joint surface diameters to confirm the results. These restricted analyses demonstrate broadly the same pattern of results, the main difference being that the temporal trend in inferred lean mass is no longer significant, perhaps as a result of small sample size. This gives confidence in our approach and findings (see Supplementary Information online for these results and further discussion of our skeletal proxies for lean mass).

As stature is a major determinant of absolute lean mass, we analysed geographic and temporal patterns in bone breadths relative to long bone lengths, as a proxy for stature. Limb bone lengths are widely used to estimate stature (e.g.^90,91^) and give relatively reliable results (standard errors of estimates 2–4 cm). The relationship between bone lengths and total stature varies temporally and geographically between populations due to variation in body proportions^92^, as well as between individuals. At present it is unclear whether available equation sets are broadly applicable to South Asian skeletons^33,93^. The same problem applies to many of the other populations in the worldwide dataset. Therefore limb bone lengths were used as a stature proxy. It is widely acknowledged that lower limb bones are better indicators of stature than upper limb bones, and that variation in torso length also contributes to stature variation^94^. As limb bone lengths show greater sensitivity to environmental conditions than trunk length^95^ they are likely to be the most variable component of stature, and thus a reliable proxy for stature variation. Furthermore, upper limb long bones are widely used in stature estimation where lower limb bones are unavailable as they are still reliable estimators of stature. Therefore we consider our approach to be justified in order to make optimal use of the limited available data.

To further maximise use of the limited available data, we calculated sex-specific z-scores for each long bone measurement separately, and then averaged them across long bones within individuals. Z-scores express measurements in standard deviation units, and permit measurements of differing magnitudes (e.g., from different skeletal elements) to be directly combined or compared. The use of sex-specific scores also allowed the sexes to be pooled. The worldwide dataset was used for the calculation of z-scores to prevent small sample sizes resulting in stochastic variation in the z-scores. For each individual skeleton, the mean of the available bone breadth z-scores and the mean of the available bone length z-scores were taken to represent the individual’s lean mass and stature respectively. To be included in the dataset, an individual required at least one length and one breadth measurement. While the combination of variables into a geometric mean to represent size is more common in anthropology, the high frequency of missing data in the sample made this approach unreliable and the z-scores were preferred to maximise statistical power and permit the combination of data from fragmentary individuals of both sexes.

### Statistical analyses

Analyses were conducted in SPSS for Windows v. 25.0 (IBM Corp., Chicago, USA) with p ≤ 0.05 considered statistically significant. The relationship between inferred lean mass and stature among South Asian skeletons relative to the worldwide dataset was assessed visually using scatterplots. An RMA regression line was fitted to the full sample in order to characterise the relationship between length and breadth z-scores, and to demonstrate when individuals had greater or less inferred lean mass relative to stature. RMA regression was chosen since the aim of this analysis was to describe the relationship between the variables, without assuming the direction of causation or the aim of producing prediction equations, and because both variables were measured with error^79,96^.

Temporal trends in South Asian inferred lean mass were assessed using ordinary least squares (OLS) regression. OLS regression was selected in this case because the dependent variable is expected to be restricted, limited or determined by the independent variable^96^. Site dates were taken from relevant publications (Supplementary Table 2). Dating methods varied widely by site, and only included direct radiometric dating of the remains themselves or their immediate contexts in a minority of cases. As most sites were dated based on cultural associations with reference to established regional chronologies, published date ranges were used and no attempt was made to re-calibrate published radiocarbon dates, which are few for any one site and unlikely to represent the full range of ages of the associated skeletons. While this may introduce some error when trying to identify temporal patterns in lean mass and stature, longer-term trends should still be evident. For analyses, site date ranges were converted to years before present and the midpoint of any range taken as the representative date for that sample.

Within the South Asian sample, mean individual bone breadth z-score was regressed on site date, adjusting for length z-score, latitude and longitude. Stature is known to decrease from north to south and west to east in South Asia^71^, so latitude and longitude were included in the initial models to account for potential geographical variation in height. Longitude was subsequently removed as it was not statistically significant. To assess temporal trends in stature, we regressed length z-score on latitude, and plotted the standardised residual against site date. We did not fit a line to the length z-score data since linear, curvilinear or LOWESS regression methods did not provide a visually convincing fit to the data across the full 11,000 years (that is, while the models could be fitted, plotting the resulting models onto the data did not provide a satisfactory correspondence between the model and the data suggesting the models were not appropriate). OLS regression was used to assess the trend in length z-score over the last 5,000 years where a straight line provided a convincing fit, and a two-tailed t-test was used to compare mean length z-scores before and after the change observed at around 5,000 years ago.

## Supplementary information

Supplementary Information

Dataset 1

## Data Availability

All data generated or analysed during this study are included in this published article (and its Supplementary Information Files). The original Goldman Dataset is freely available online at https://web.utk.edu/~auerbach/GOLD.htm.
