# Supplementary material for: Ancient origins of low lean mass among South Asians and implications for modern type 2 diabetes susceptibility
Source: Sci Rep. 2019 Jul 19;9:10515. doi: 10.1038/s41598-019-46960-9 (PMC6642207; doi:10.1038/s41598-019-46960-9)
Supplement: Supplementary file 1 — Supplementary Information [file 41598_2019_46960_MOESM1_ESM.docx]

**Ancient origins of low lean mass among South Asians and implications for modern type 2 diabetes susceptibility**

Emma Pomeroy, Veena Mushrif-Tripathy, Tim J. Cole, Jonathan C.K. Wells and Jay T. Stock

**Supplementary Information**

**Supplementary Table 1. Details of ordinary least squares regression of mean individual bone breadth z-score on date midpoint, adjusting for mean individual length z-score and latitude.** Each successive model includes the predictors in the preceding rows. SEE = standard error of estimate; B = regression coefficient in final model; SE = standard error; β = standardised coefficient in final model; df = degrees of freedom.

| **Predictor (s)** | **R** | **R^2^** | **Adjusted R^2^** | **SEE** | **B (SE)** | **β** | **R^2^ change** | **F change** | **df1** | **df2** | **p (F change)** |
| --- | --- | --- | --- | --- | --- | --- | --- | --- | --- | --- | --- |
| Mean length z-score | 0.66 | 0.43 | 0.43 | 0.70 | 0.47 (0.05) | 0.61 | 0.43 | 146.35 | 1 | 195 | <0.001 |
| + latitude  (decimal degrees) | 0.73 | 0.53 | 0.52 | 0.63 | 0.05 (0.01) | 0.33 | 0.12 | 43.7 | 1 | 194 | <0.001 |
| + date midpoint (thousand years) | 0.73 | 0.55 | 0.54 | 0.63 | 0.04 (0.02) | -0.13 | 0.01 | 5.0 | 1 | 193 | 0.03 |

**Supplementary Table 2. Sample composition for South Asian skeletal data.** Sites are grouped approximately by date and subsistence type. The Mesolithic samples represent hunter-gatherers, but overlap temporally with some Prehistoric sites**.** Lat. = Latitude; Long. = Longitude; BP = years before present; cal = calibrated; OSL = Optically Stimulated Luminescence; AMS = Accelerator Mass Spectrometer radiocarbon dating; C14 = standard or unspecified radiocarbon dating; TL = Thermoluminescence.

| **Site** | **Country** | **State** | | **Lat.** | | | **Long.** | **n** | **Date** | **Date mid-point (BP)** | | **Data source** | | **Date type** | | **Date source** |
| --- | --- | --- | --- | --- | --- | --- | --- | --- | --- | --- | --- | --- | --- | --- | --- | --- |
| **Mesolithic**  **(11,000-4,300 BP)** |  | |  | |  | |  |  |  | |  | |  |  |  | |
| Bellan Bandi Palassa | Sri Lanka | Sabaragamuva Province | | 6.5 | | | 80.8 | 2 | 11,150 – 12,250 cal BP, 11,620 – 12,050 cal BP | 11,000 | | ^1^ | | AMS | | ^2^ |
| Sarai Nahar Rai | India | Uttar Pradesh | | 25.8 | | | 81.8 | 8 | 10,050+/-110 BP | 10,000 | | ^3^ | | C14 | | Sharma (1973); Sharma and Sharma (1978) in ^4^ |
| Fa Hien | Sri Lanka | Western Province | | 6.7 | | | 80.2 | 1 |  | 10,400 | | JTS/EP | | AMS | | T. Higham pers. comm. 2018 |
| Damdama | India | Uttar Pradesh | | 26.2 | | | 82.2 | 18 | 8865 (DDM12), 8640 (DDM 36a) +/-65 cal BP | 8,750 | | ^4^ | | AMS | | ^5, 6^ |
| Kuragala | Sri Lanka | Sabaragamuwa  Province | | 6.6 | | | 80.9 | 1 |  | 7,000 | | JTS/EP | | AMS | | M. Petraglia pers. comm. 2014 |
| Mahadaha | India | Uttar Pradesh | | 26.0 | | | 82.2 | 17 | 6320 +/-80 cal BP | 6,300 | | ^7^ | | AMS | | ^8^ |
| Bhimbetka Mesolithic | India | Madhya Pradesh | | 22.9 | | | 77.6 | 2 | 10,000-2,500 BP | 6,250 | | VMT-EP | | Phasing, C14 | | ^9^ |
| Mini-athiliya | Sri Lanka | Southern Province | | 6.1 | | | 80.9 | 1 | 3610 ± 40 BP, 3680 ± 40 BP | 5,650 | | ^10^ | | C14, | | ^10^ |
| Baghai Khor | India | Uttar Pradesh | | 24.8 | | | 82.1 | 1 | 3000-2000 BC | 4,500 | | ^11^ | | Phasing | | ^11^ |
| Langhnaj | India | Gujarat | | 23.3 | | | 72.5 | 3 | 2440-2160 cal BC | 4,300 | | ^12^ | | C14 | | ^13^ |
| **Prehistoric**  **(4,500-2,000 BP)** |  | |  | |  | |  |  |  | |  | |  |  |  | |
| Nagarjunakonda Neolithic | India | Andhra Pradesh | | | | 16.5 | 79.2 | 1 | 2000-1000 BC | 4,500 | | ^14^ | | Phasing | | ^14^ |
| Rakhighari | India | Haryana | | | | 29.3 | 76.1 | 2 | 4600-4300 BP | 4,450 | | VMT-EP | | C14 | | ^15^ |
| Farmana | India | Haryana | | | | 29.0 | 76.3 | 2 | 2600-2000 BC | 4,300 | | VMT-EP | | Phasing | | ^16^ |
| Harappa | Pakistan | Punjab | | | | 30.6 | 72.9 | 27 | 2550-2030 BC | 4,300 | | ^17^ | | Phasing, C14 | | ^16^ |
| Lothal Mature Harappan | India | Gujarat | | | | 22.5 | 72.3 | 4 | 2200-1900 BC | 4,000 | | ^18^ | | Phasing, C14 | | ^19^ |
| Burzahom | India | Jammu and Kashmir | | | | 34.2 | 74.9 | 6 | 2300-1500 BC | 3,900 | | ^20^ | | Phasing, C14 | | ^20, 21^ |
| Kaothe | India | Maharashtra | | | | 21.0 | 74.3 | 1 | 2000-1800 BC | 3,900 | | ^22^ | | C14 | | ^23^ |
| Lothal Late Harappan | India | Gujarat | | | | 22.5 | 72.3 | 1 | 1900-1600 BC | 3,750 | | ^18^ | | Phasing, C14 | | ^19^ |
| Rupar | India | Punjab | | | | 30.9 | 76.6 | 1 | 2100-1400 BC | 3,750 | | ^24^ | | Phasing | | ^24^ |
| Tenner | India | Andhra Pradesh | | | | 16.5 | 80.8 | 1 | 1200-300 BC | 3,750 | | ^25^ | | Phasing | | ^25^ |
| Veerabyina Kunta | India | Andhra Pradesh | | | | 17.3 | 82.1 | 1 | 1200-300 BC | 3,750 | | ^25, 26^ | | Phasing | | ^25^ |
| T. Narsipur | India | Karnataka | | | | 12.2 | 76.9 | 1 | 3500 BP | 3,500 | | ^27^ | | C14 | | ^28^ |
| Chinnamarur | India | Andhra Pradesh | | | | 16.0 | 78.1 | 2 | 2000-700 BC | 3,350 | | VMT-EP | | Phasing | | ^29^ |
| Nevasa | India | Maharashtra | | | | 19.6 | 74.9 | 2 | 1500-1000 BC | 3,250 | | VMT-EP | | Phasing | | ^30^ |
| Bhagwanpura | India | Haryana | | | | 30.1 | 77.0 | 1 | 1400–1000 BC | 3,200 | | ^31^ | | TL, ceramic | | ^31^ |
| Chandoli | India | Maharashtra | | | | 19.0 | 74.0 | 1 | 1400-1050 BC | 3,200 | | ^27^ | | C14 | | ^32^ |

***Supplementary Table 2 cont.***

| **Site** | **Country** | **State** | | | **Lat.** | **Long.** | **N** | **Date** | **Date mid-point (BP)** | | **Data source** | | **Date type** | | **Date source** |
| --- | --- | --- | --- | --- | --- | --- | --- | --- | --- | --- | --- | --- | --- | --- | --- |
| **Prehistoric**  **(4,500-2,000 BP) cont.** |  | |  |  | |  |  |  | |  | |  |  |  | |
| Pochampad | India | Andhra Pradesh | | | 18.8 | 78.3 | 2 | 300 BC-AD 50 | 3,125 | | ^33^ | | Phasing | | ^33^ |
| Inamgaon | India | Maharashtra | | | 18.6 | 74.5 | 5 | 1400-700 BC | 3,050 | | VMT-EP | | Phasing | | ^34^ |
| Pandu Rajar Dhibi | India | West Bengal | | | 23.6 | 87.7 | 5 | 1012+/- 120 BC | 3,000 | | ^35^ | | C14 | | Dasgupta (1964) in ^35^ |
| Piklihal Upper Neolithic | India | Karnataka | | | 16.0 | 76.5 | 1 | 1250-650 BC | 2,950 | | ^36^ | | Phasing | | ^37^ |
| Abhaipur | India | Uttar Pradesh | | | 28.3 | 79.8 | 1 | 1200-400 BC | 2,800 | | ^38^ | | Phasing | | ^38^ |
| Nagarjunakonda Late | India | Andhra Pradesh | | | 16.5 | 79.2 | 1 | 800-300 BC | 2,550 | | ^14^ | | Phasing | | ^14^ |
| Yeleswaram | India | Andhra Pradesh | | | 16.5 | 79.2 | 2 | 200-299 BC | 2,250 | | ^39^ | | Phasing | | ^39^ |
| Kodumanal | India | Tamil Nadu | | | 11.1 | 77.5 | 3 | 200 BC-AD 200 | 2,000 | | VMT-EP | | Phasing | | ^40^ |
| **Historic (2,100-200 BP)** |  | |  | |  |  |  |  | |  | |  |  |  | |
| Nevasa Indo-Roman | India | Maharashtra | | | 19.6 | 74.9 | 1 | 50 BC- AD 200 | 2,100 | | VMT-EP | | Phasing | | ^30^ |
| Padri | India | Gujarat | | | 22.4 | 72.6 | 1 | 100 BC- AD 100 | 2,000 | | VMT-EP | | Phasing | | ^41^ |
| Kuntasi | India | Gujarat | | | 22.9 | 70.6 | 2 | 8^th^ - 18^th^ century AD | 700 | | VMT-EP | | Phasing | | ^42^ |
| Bagor Historic | India | Rajasthan | | | 25.4 | 74.4 | 1 | 1200-1700 AD | 550 | | ^43^ | | Phasing | | ^43, 44^ |
| Balupur | India | West Bengal | | | 25.1 | 88.1 | 7 | 1400-1700 AD | 450 | | VMT-EP | | OSL, ceramics | | ^45^ |
| Leshmi | India | Nagaland | | | 25.5 | 94.2 | 2 | Mid-17^th^ century AD | 350 | | VMT-EP | | Phasing | | ^46^. |
| Jotsoma | India | Nagaland | | | 25.7 | 94.1 | 4 | AD 1650-1950 | 200 | | VMT-EP | | Phasing | | ^47^ |
| **Modern (100-40 BP)** |  | |  | |  |  |  |  | |  | |  |  |  | |
| Veddah | Sri Lanka |  | | | 7.5 | 81.5 | 11 | c. 1880-1935 | 100 | | ^48^ | | Acquisition history | | ^48, 49^ |
| University of Toronto Teaching collection | India | West Bengal | | | 22.6 | 88.4 | 40 | ~ AD 1960 | 40 | | EP | | Acquisition history | | S. Pfeiffer (pers. comm. August 2016) |

**Supplementary Information 1.1: Additional discussion on inferring lean mass from the skeleton**

The use of bone shaft dimensions as a proxy for lean mass may be a limitation of our study, as these dimensions are known to be influenced by activity, body mass changes during life and age (see Methods). Mesolithic hunter-gatherer populations were often more mobile than their agricultural successors ^33, 50, 51, 52, 53^, but the transition from high mobility to agricultural labour may have had unknown effects on musculature. Some evidence suggests that hunter-gatherers would have been more muscular (i.e., have had greater lean mass) and have had greater bone diameters resulting from elevated activity compared with agriculturalists: greater muscular forces among Mesolithic individuals have been inferred for South Asia from the greater development of muscle insertions compared with agriculturalists ^6, 22, 54, 55, 56^. We might therefore predict that if differences in activity patterns were confounding trends in lean mass, the Mesolithic individuals would have had elevated relative bone breadth z-scores compared with the later populations in our dataset. In fact, there was no statistical trend in our proxy for relative lean mass, and if anything the Mesolithic individuals had lower inferred lean mass than later populations (Figure 4 and Supplementary Figure 4). Thus we are confident that low lean mass traces back at least 11,000 years in South Asia. If activity has affected the results, this would suggest an increase in relative lean mass since the Mesolithic, which would still be inconsistent with alternative hypotheses regarding the origins of low lean mass in the region. Moreover, we repeated our analyses using only joint surface breadths to calculate our lean mass proxy, and the fact that results remain unchanged (Figures 1 and 2, Supplementary Table 3) gives additional confidence in our findings.

It might also be argued that our bone breadth proxies for lean mass reflect variation in body breadth rather than lean mass per se. However we note that stature and body (bi-iliac) breadth (BIB) are considered the major determinants of body mass in human populations ^57, 58, 59^, the largest component of body mass is lean mass ^60^. While stature and BIB do not fully account for variation in body mass, particularly in populations with broad shoulders ^58^ or heavily muscular elite athletes (shot putters, weightlifters etc.) ^59^, they are good predictors of body mass across a wide range of populations (standard errors of estimate 3.7 - 4.0 kg: ^58^). Although not stated explicitly, presumably the samples used to derive these estimation equations were largely non-obese since the studies ^57, 58, 61^ aimed to demonstrate relationships between climate, stature, body breadth and body mass, so we might expect stature and BIB to be closely related to lean mass. Small BIB is considered a long-term adaptation to heat stress in humans ^57, 61^ and we can speculate that small BIB is associated with smaller trunk volume and thus lower organ mass. While our data cannot exclude the possibility that the low lean mass of South Asians is decoupled from BIB, anecdotally other populations with a similar low lean mass phenotype also have relatively low BIB (e.g., Australian males average 25.6 cm and Indian males 25.9 cm in the data reproduced in Ruff ^57^), In contrast, populations known to have high lean mass despite inhabiting a warm climate have high BIB (e.g. Polynesians ^62^: male BIB = 29.4 cm in Ruff ^57^). While further research into the interrelationship between low lean mass and body breadth is clearly needed, since body breadth, body mass and lean mass are inter-related, our results remain a reliable indication of trends in lean mass.

**
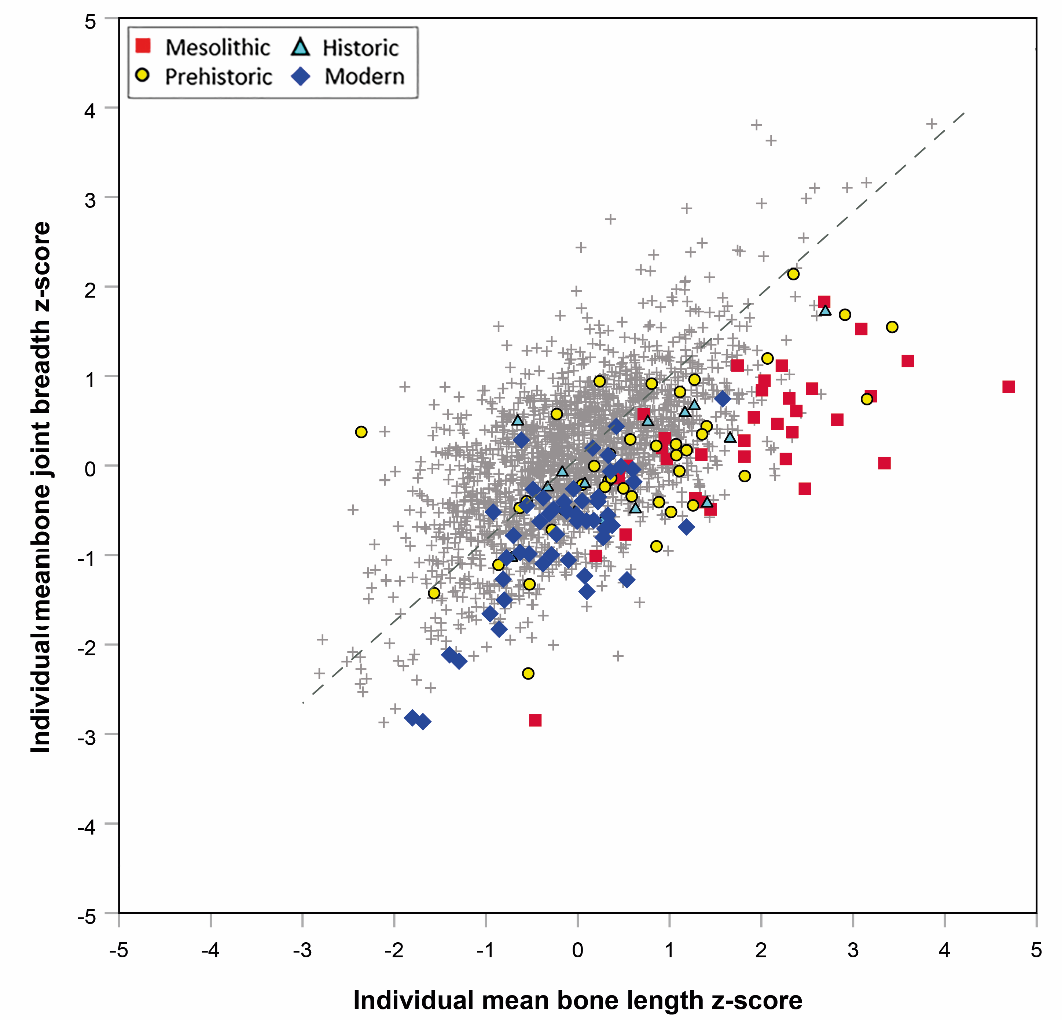
**

**Supplementary Figure 1. Plot of individual mean joint breadth z-score against bone length z-score in South Asian archaeological and modern skeletons from the last 11,000 years (n = 139) compared with a worldwide sample of terminal Pleistocene and Holocene humans (n = 1,884: grey crosses)** demonstrating that South Asians throughout the study period typically had low lean mass (joint breadth z-score) relative to stature (bone length z-score). Reduced major axis regression line fitted to the whole dataset shown as grey dashed line.

**Supplementary Table 3. Details of ordinary least squares regression of mean individual bone joint surface breadth z-score on date midpoint, adjusting for mean individual length z-score and latitude (n = 139).** Each successive model includes the predictors in the preceding rows. SEE = standard error of estimate; B = regression coefficient in final model; SE = standard error; β = standardised coefficient in final model; df = degrees of freedom.

| **Predictor (s)** | **R** | **R^2^** | **Adjusted R^2^** | **SEE** | **B (SE)** | **β** | **R^2^ change** | **F change** | **df1** | **df2** | **p (F change)** |
| --- | --- | --- | --- | --- | --- | --- | --- | --- | --- | --- | --- |
| Mean length z-score | 0.76 | 0.57 | 0.57 | 0.72 | 0.66 (0.06) | 0.76 | - | 181.5 | 1 | 137 | <0.001 |
| + latitude  (decimal degrees) | 0.78 | 0.61 | 0.60 | 0.69 | 0.04 (0.01) | 0.21 | 0.03 | 12.4 | 1 | 136 | 0.001 |
| + date midpoint (thousand years) | 0.78 | 0.61 | 0.61 | 0.69 | -0.04 (0.02) | -0.12 | 0.01 | 3.0 | 1 | 135 | 0.09 |


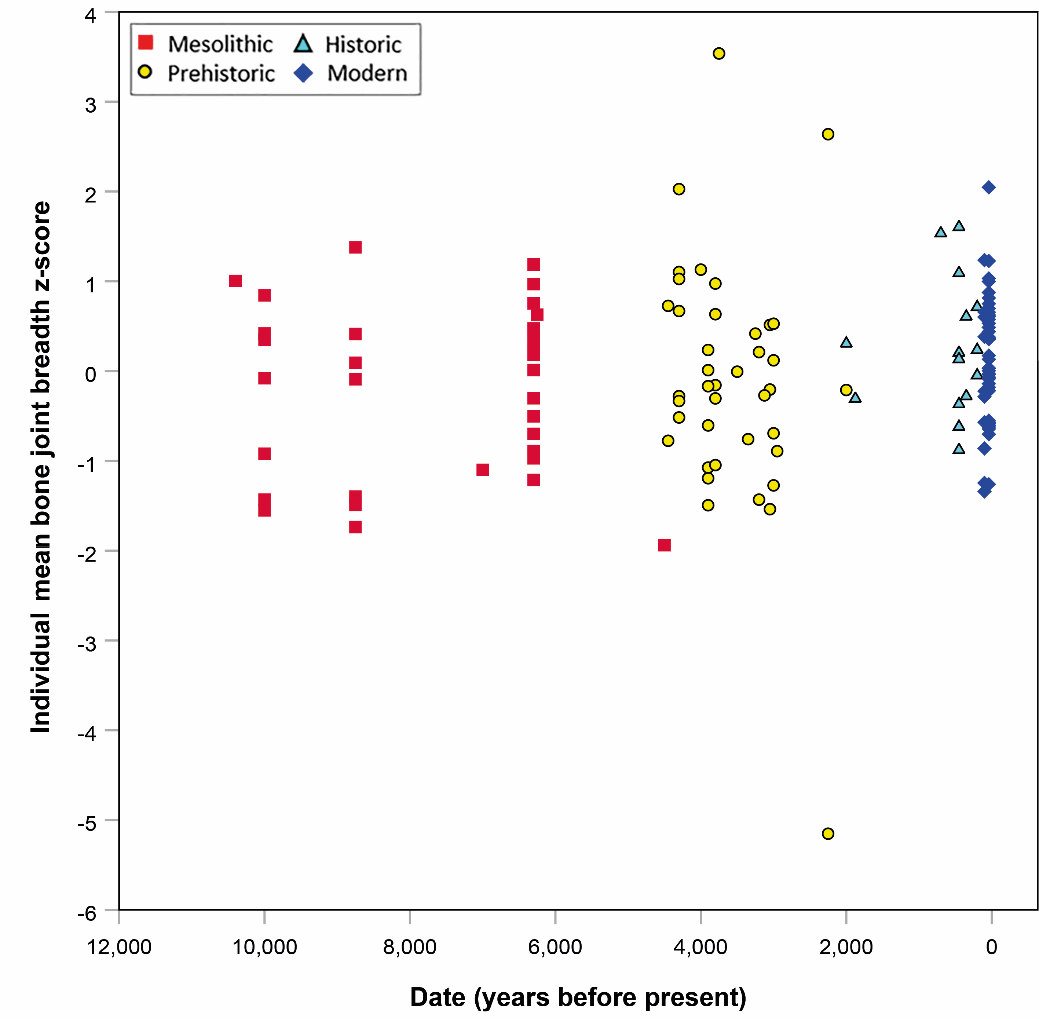


**Supplementary Figure 2. Individual mean joint breadth z-score (adjusted for latitude and bone length z-score) plotted against date of site, illustrating no temporal trend in relative lean mass among South Asians (n = 139) over the last 11,000 years**.

**References**

1. Kennedy, K.A.R. Human skeletal remains from Ceylon, with an analysis of the island's prehistoric and contemporary populations. *Bull. Br. Mus.(Nat. Hist.): Geol.* **11**, 135-213 (1965).

2. Simpson, I., Kourampas, N. & Perera, H.N. Bellan-bandi Palassa, Sri Lanka: Formation processes of a Mesolithic open-air site identified through thin section micromorphology. *Archaeologia* **4**, 3-18 (2008).

3. Kennedy, K.A.R., Lovell, N.C. & Burrow, C.B. *Mesolithic human remains from the Gangetic Plain: Sarai Nahar Rai*. South Asia Program, Cornell University (1986).

4. Lukacs, J.R. & Pal, J.N. Skeletal variation among Mesolithic People of the Ganga Plains: New evidence of habitual activity and adaptation to climate. *Asian Perspect.* **42**, 329-351 (2003).

5. Lukacs, J., Pal, J. & Misra, V. Chronology and diet in Mesolithic North India: A preliminary report of the AMS C 14 dates, 15 C isotopes, and their significance in *Colloquia of the International Congress of Prehistoric and Protohistoric Sciences* (1997).

6. Lukacs, J.R. Mesolithic Foragers of the Ganges Plain In *A Companion to South Asia in the Past* (eds. Robbins-Schug, G. & Walimbe, S.R.). John Wiley & Sons, Inc (2016).

7. Kennedy, K.A.R.*, et al.* *Human skeletal remains from Mahadaha: a Gangetic mesolithic site*. South Asia Program, Cornell University (1992).

8. Chattopadhyaya, U.C. Settlement pattern and the spatial organization of subsistence and mortuary practices in the Mesolithic Ganges Valley, North-Central India. *World Archaeol.* **27**, 461-476 (1996).

9. Kennedy, K.A.R., Misra, V.N. & Burrow, C.B. Dental Mutilations from Prehistoric India. *Curr. Anthropol.* **22**, 285-286 (1981).

10. Kulatilake, S., Perera, N., Deraniyagala, S. & Perera, J. The discovery and excavation of a human burial from the Mini-athiliya Shell Midden in southern Sri Lanka. *Ancient Asia* **5**, 108 (2014).

11. Kennedy, K.A.R. Porotic hyperostosis on human remains from mesolithic Baghai Khor. *Bulletin of the Deccan College Post Graduate and Research Institute* **49**, 183-198 (1990).

12. Ehrhardt, S. & Kennedy, K.A. *Excavations at Langhnaj: 1944-63, Pt. 3: the Human Remains*. Deccan College, Postgraduate and Research Institute (1965).

13. Possehl, G.L. Harappans and hunters: economic interaction and specialization in prehistoric India in *Forager-Traders in South and Southeast Asia: Long-Term Histories* (eds. Morrison, K.D. & Junker, L.L.). Cambridge University Press (2002).

14. Gupta, P., Dutta, P.C. & Basu, A. A study of the Nagarjunakonda skeletons in *Ancient human remains*(eds. Gupta, P., Basu, A. & Dutta, P.C.). Anthropological Survey of India (1970).

15. Nath, A., Walimbe, S.R., Garge, T.M., Mushrif-Tripathy, V., Dehuri, R. & Malik, A. Harappan interments at Rakhigarhi, Haryana. *Man Environ.* **40**, 9-32 (2015).

16. Valentine, B.*, et al.* Evidence for Patterns of Selective Urban Migration in the Greater Indus Valley (2600-1900 BC): A Lead and Strontium Isotope Mortuary Analysis. *PLoS One* **10**, e0123103 (2015).

17. Gupta, P., Dutta, P.C. & Basu, A. *Human Skeletal Remains from Harappa*. Anthropological Survey of India, Government of India (1962).

18. Sarkar, S.S. Human skeletal remains from Lothal in *Lothal - A Harappan port town (1955-1962)* (ed. Rao, S.R.). Archaeological Survey of India (1985).

19. Rao, S.R. *Lothal - A Harappan port town (1955-1962)*. Archaeological Survey of India (1985).

20. Basu, A. & Pal, A. *Human remains from Burzahom*. Anthropological Survey of India, Govt. of India (1980).

21. Sharma, A.K. Neolithic human burials from Burzahom, Kashmir. *J. Oriental Inst.* **16**, 240-242 (1967).

22. Walimbe, S.R. Human Skeletal Remains in *Excavations at Kaothe* (eds. Dhavilakar, M.K., Shinde, V. & Atre, S.). Deccan College Post-Graduate and Research Institute (1990).

23. Dhavilakar, M.K., Shinde, V. & Atre, S. Excavations at Kaothe. Deccan College Post-Graduate and Research Institute (1990).

24. Dutta, P.C., Pal, A., Gupta, P. & Dutta, B.C. *Ancient human remains from Rupar*. Anthropological Survey of India (1987).

25. Walimbe, S.R., Caldwell-Ott, P.C. & Kennedy, K.A.R. Hitherto undescribed megalithic burials from South Asia: The skeletal record. *Man Environ.* **36**, 31-70 (2011).

26. Gupta, P. & Dutta, P.C. Human remains excavated from megaliths at Yelleswaram (Andhra Pradesh). *Man in India* **42**, 19-34 (1962).

27. Malhotra, K.C. Report on the human skeleton remains from Neolithic T. Narsipur (Mysore state) in *Report on the excavations at T. Narsipur* (ed. Seshadri, M.). Government of Mysore (1971).

28. Seshadri, M. *Report on the excavations at T. Narsipur*. Government of Mysore (1971).

29. Walimbe, S.R. Population movements in the Indian subcontinent during the protohistoric period: Physical anthropological assessment in *The Evolution and History of Human Populations in South Asia: Inter-disciplinary Studies in Archaeology, Biological Anthropology, Linguistics and Genetics* (eds. Petraglia, M.D. & Allchin, B.). Springer Netherlands (2007).

30. Mushrif-Tripathy, V., Walimbe, S.R. *Human skeletal remains from Chalcolithic Nevasa: osteobiographic analysis*. John and Erica Hedges Ltd. (2006).

31. Joshi, J.P. *Excavation at Bhagwanpura, 1975-76, and other explorations & excavations, 1975-81, in Haryana, Jammu & Kashmir, and Punjab* (1993).

32. Deo, S.B. & Ansari, Z.D. *Chalcolithic Chandoli: Report on the excavations at Chandoli 1960*. Deccan College Postgraduate and Research Institute (1965).

33. Kennedy, K.A.R. Megalithic Pochampad: The skeletal biology and archaeological context of an Iron Age site in Andhra Pradesh, India. *Asian Perspect.* **41**, 103-128 (2002).

34. Lukacs, J.R. & Walimbe, S.R. *Excavations at Inamgaon Volume II: The Physical Anthropology of Human Skeletal Remains; Part 1: An Osteobiographic Analysis*. Deccan College Post Graduate and Research Institute (1986).

35. Gupta, P. & Lal, A. Human skeletal materials excavated at Pandu Rajar Dhibi. *Bull. Anthropol. Survey India* **29**, 127-202 (1970 (1977)).

36. Ayer, A.A. Appendix 1. Report on human skeletal remains excavated at Piklihal, near Mudgal in *Piklihal excavations* (ed. Allchin, F.R.). Government of Andhra Pradesh (1960).

37. Allchin, F.R. *Piklihal excavations*. Government of Andhra Pradesh (1960).

38. Mushrif-Tripathy, V., Misra, A., Arora, U.P. & Walimbe SR. Anthropological studies on human skeletons from the PGW site of Abhaipur (District Pilibhit), U.P. *Man Environ.* **33**, 80-100 (2008).

39. Kennedy, K. Yeleswaram revisited: the skeletal record. *Man Environ.* **35**, 35-57 (2000).

40. Mushrif-Tripathy, V., Rajan, K. & Walimbe, S.R. *Megalithic builders of South India: archaeo-anthropological investigations on human skeletal remains from Kodumanal*. Centre for Ancient Human Skeletal Studies (2011).

41. Walimbe, S. & Shinde, V. Anthropometric data on a human skeleton from the Early Historic levels at Padri. *Man Environ.* **20**, 43-55 (1995).

42. Walimbe, S.R. & Mushrif, V. Human skeletal remains of the historical period at Kuntasi, District Rajkot, Gujarat. *Bulletin of the Deccan College Research Institute* **58/59**, 117-139 (1998).

43. Kennedy, K.A.R. The biological anthropology of the human skeletal remains from Bagor: osteology in *Bagor and Tilwara: late Mesolithic cultures of Northwest India. Volume 1, The human skeletal remains* (eds. Lukacs, J.R., Misra, V.N., & Kennedy, K.A.R.). Deccan College Post Graduate and Research Institute (1982).

44. Misra, V.N. Bagor - A late mesolithic settlement in north-west India. *World Archaeol.* **5**, 92-110 (1973).

45. Panja, S., Nag, A.K. & Bandyopadhyay, S. *Living with Floods: Archaeology of a Settlement in the Lower Ganga Plains, C.600-1800 CE*. Ratna Sagar P. Limited (2016).

46. Mushrif-Tripathy, V. & Jamir, T. Study of two skeletons from Leshemi: A burial site in Nagaland. *Man Environ.* **36**, 71-78 (2011).

47. Mushrif-Tripathy, V., Chakraborty, K.S. & Lahiri, S. Where Are They Now? The Human Skeletal Remains from India. In: *A Companion to South Asia in the Past* (eds. Robbins-Schug, G. & Walimbe, S.R.). John Wiley & Sons, Inc (2016).

48. Osman Hill, W.C. The physical anthropology of existing Veddahs of Ceylon. Part 2. *Ceylon J. Sci. Section G: Anthropol.* **3**, 147-234 (1941).

49. Osman Hill, W.C. The physical anthropology of existing Veddahs of Ceylon. Part 1. *Ceylon J. Sci. Section G: Anthropol.* **3**, 27-144 (1941).

50. Ruff, C.B.*, et al.* Gradual decline in mobility with the adoption of food production in Europe. *Proc. Natl. Acad. Sci. USA* **112**, 7147-7152 (2015).

51. Ryan, T.M. & Shaw, C.N. Gracility of the modern Homo sapiens skeleton is the result of decreased biomechanical loading. *Proc. Natl. Acad. Sci. USA* **112**, 372-377 (2015).

52. Ruff, C.B., Larsen, C.S. & Hayes, W.C. Structural changes in the femur with the transition to agriculture on the Georgia coast. *Am. J. Phys. Anthropol.* **64**, 125-136 (1984).

53. Brock, S.L. & Ruff, C.B. Diachronic patterns of change in structural properties of the femur in the Prehistoric American Southwest. *Am. J. Phys. Anthropol.* **75**, 113-127 (1988).

54. Kennedy, K.A.R. Skeletal adaptations of Mesolithic hunter-foragers of North India: Mahadaha and Sarai Nahar Rai compared in *The Prehistory of Asia and Oceania* (eds. Afanas'ev, G., Cleuziou, S., Lukacs, J.R. & Tosi, M.). ABACO Edizioni (1996).

55. Lukacs, J.R. Human biological diversity in ancient India: Dr Irawati Karve and contemporary issues in biological anthropology in *Anthropology for archaeology: proceedings of the Professor Irawati Karve birth centenary seminar* (eds. Walimbe, S.R., Joglekar, P.P. & Basa, K.K.). Deccan College Post-Graduate and Reseaech Institute (2007).

56. Kennedy, K.A.R. Climatic events and environmental adaptations relating to the Mesolithic hominids of the Gangetic Plain. *Quat. Int.* **192**, 14-19 (2008).

57. Ruff, C.B. Morphological adaptation to climate in modern and fossil hominids. *Am. J. Phys. Anthropol.* **37**, 65-107 (1994).

58. Ruff, C.B., Niskanen, M., Junno, J.-A. & Jamison, P. Body mass prediction from stature and bi-iliac breadth in two high latitude populations, with application to earlier higher latitude humans. *J. Hum. Evol.* **48**, 381-392 (2005).

59. Ruff, C.B. Body mass prediction from skeletal frame size in elite athletes. *Am. J. Phys. Anthropol.* **113**, 507-517 (2000).

60. Wells, J.C.K. Maternal capital and the metabolic ghetto: An evolutionary perspective on the transgenerational basis of health inequalities. *Am. J. Hum.Biol.* **22**, 1-17 (2010).

61. Ruff, C.B. Climate and body shape in hominid evolution. *J. Hum. Evol.* **21**, 81-105 (1991).

62. Wells, J.C.K. Ecogeographical associations between climate and human body composition: Analyses based on anthropometry and skinfolds. *Am. J. Phys. Anthropol.* **147**, 169-186 (2012).
